# Very Delayed Remote Ischemic Post-conditioning Induces Sustained Neurological Recovery by Mechanisms Involving Enhanced Angioneurogenesis and Peripheral Immunosuppression Reversal

**DOI:** 10.3389/fncel.2018.00383

**Published:** 2018-10-29

**Authors:** Thorsten R. Doeppner, Bozena Zechmeister, Britta Kaltwasser, Fengyan Jin, Xuan Zheng, Arshad Majid, Vivek Venkataramani, Mathias Bähr, Dirk M. Hermann

**Affiliations:** ^1^Department of Neurology, University Medical Center Göttingen, Göttingen, Germany; ^2^Department of Neurology, University Duisburg-Essen Medical School, Essen, Germany; ^3^Cancer Center, The First Hospital of Jilin University, Changchun, China; ^4^Sheffield Institute for Translational Neuroscience, University of Sheffield, Sheffield, United Kingdom; ^5^Department of Hematology & Oncology, University Medical Center Göttingen, Göttingen, Germany; ^6^Institute of Pathology, University Medical Center Göttingen, Göttingen, Germany

**Keywords:** focal cerebral ischemia, remote ischemic post-conditioning, neuroprotection, neuroregeneration, immune response, proteasome

## Abstract

Ischemic conditioning is defined as a transient and subcritical period of ischemia integrated in an experimental paradigm that involves a stimulus of injurious ischemia, activating endogenous tissue repair mechanisms that lead to cellular protection under pathological conditions like stroke. Whereas ischemic pre-conditioning is irrelevant for stroke treatment, ischemic post-conditioning, and especially non-invasive remote ischemic post-conditioning (rPostC) is an innovative and potential strategy for stroke treatment. Although rPostC has been shown to induce neuroprotection in stroke models before, resulting in some clinical trials on the way, fundamental questions with regard to its therapeutic time frame and its underlying mechanisms remain elusive. Hence, we herein used a model of non-invasive rPostC of hind limbs after cerebral ischemia in male C57BL6 mice, studying the optimal timing for the application of rPostC and its underlying mechanisms for up to 3 months. Mice undergoing rPostC underwent three different paradigms, starting with the first cycle of rPostC 12 h, 24 h, or 5 days after stroke induction, which is a very delayed time point of rPostC that has not been studied elsewhere. rPostC as applied within 24 h post-stroke induces reduction of infarct volume on day three. On the contrary, very delayed rPostC does not yield reduction of infarct volume on day seven when first applied on day five, albeit long-term brain injury is significantly reduced. Likewise, very delayed rPostC yields sustained neurological recovery, whereas early rPostC (i.e., <24 h) results in transient neuroprotection only. The latter is mediated via heat shock protein 70 that is a well-known signaling protein involved in the pathophysiological cellular cascade of cerebral ischemia, leading to decreased proteasomal activity and decreased post-stroke inflammation. Very delayed rPostC on day five, however, induces a pleiotropic effect, among which a stimulation of angioneurogenesis, a modulation of the ischemic extracellular milieu, and a reversal of the stroke-induced immunosuppression occur. As such, very delayed rPostC appears to be an attractive tool for future adjuvant stroke treatment that deserves further preclinical attention before large clinical trials are in order, which so far have predominantly focused on early rPostC only.

## Introduction

With the recent success of endovascular strategies to avoid stroke induced brain injury (Campbell et al., 2015; Goyal et al., 2015), treatment paradigms for stroke patients have significantly changed. Nevertheless, restricted eligibility of patients for both endovascular treatment and especially systemic thrombolysis requests for novel adjuvant treatment paradigms (Lees et al., 2010). In this context, enhancing the endogenous resistance and regenerative capacity of the ischemic brain appears to be very attractive, thus avoiding the need of neuroprotective drug delivery or stem cell transplantation, to name but a few experimental concepts.

Ischemic conditioning is an experimental strategy that has been studied for decades, using a variety of animal models including but not limited to stroke models (Zhang et al., 2003; Ding et al., 2004; Gidday, 2006; Davis et al., 2007; Xie et al., 2013; Badawi et al., 2015). As such, ischemic conditioning is defined as a transient and subcritical period of ischemia embedded in an experimental setup where ischemic conditioning can be induced before (pre-conditioning), during (per-conditioning) or after (post-conditioning) injurious ischemia. Although studies on ischemic pre-conditioning in stroke models have given new insights into the pathogenesis of stroke, involving a plethora of signaling molecules such as the nuclear factor (NF)-κB, hypoxia-inducible factor (HIF)-1α, tumor necrosis factor (TNF)-α, and inducible NO synthase (iNOS) (Gidday, 2006; Garcia-Bonilla et al., 2014), ischemic pre-conditioning is irrelevant from a therapeutic point of view. The scientific focus has therefore switched from ischemic pre-conditioning toward ischemic post-conditioning of the brain.

To this date, ischemic post-conditioning has been shown to be neuroprotective in a variety of experimental stroke models (Gao et al., 2008; Xing et al., 2008; Anrather and Hallenbeck, 2013; Xie et al., 2013), albeit precise signaling cascades involved in this neuroprotective process have not been shown for the majority of studies. Recently, our group has not only demonstrated that direct ischemic post-conditioning is neuroprotective in a model of transient focal cerebral ischemia in mice, but also that ischemic post-conditioning facilitates transplantation of neural progenitor cells (NPCs) leading to sustained neurological recovery (Doeppner et al., 2017). The latter is a consequence of pleiotropic mechanisms, among which stabilization of the blood-brain barrier, reduction of leukocyte infiltration into the brain, and reduction of oxidative stress levels play pivotal roles. Despite positive proof-of-concept studies on cerebral ischemic post-conditioning, the latter are unlikely to be translated into a real world clinical setting which would then imply induction of a second subsequent cerebral ischemia in acute stroke patients.

Whereas immediate ischemic post-conditioning of the brain itself is impractical in clinical settings for obvious reasons, the concept of remote ischemic post-conditioning (rPostC) has gained a substantial amount of attraction. Indeed, rPostC in general and hind limb rPostC in particular has been shown to be neuroprotective, albeit survival periods of these studies did not extend 2 weeks, giving insights in some underlying cellular signaling pathways only (Hasseldam et al., 2013; Li S. et al., 2015; Ren et al., 2015, 2018; Ramagiri and Taliyan, 2017a,b). Although first clinical trials with regard to rPostC in stroke-like settings are underway with indication of both a putative beneficial effect and safety (Hou et al., 2016; England et al., 2017; Wang et al., 2017; Zhao et al., 2017), further pre-clinical studies are urgently needed in order to address fundamental issues. As a matter of fact, the RECAST trial remains the only trial using rPostC as a tool for acute stroke treatment, whereas other trials focus on the impact of rPostC on conditions of chronic vessel disease only. Using a model of transient focal cerebral ischemia in mice followed by hind limb rPostC, the present study therefore addresses the therapeutic time window of rPostC beyond application time points of 24 h and further elucidates cellular mechanisms being involved in the process.

## Materials and Methods

### Experimental Animals and Induction of Transient Focal Cerebral Ischemia in Mice

All experiments were carried out according to the guidelines for the care and health of animals and were approved by local authorities. Experimental procedures followed the ARRIVE recommendations. Mice were randomly allocated to the experimental treatment groups. Surgeons and analysts were different from each other so that analysts were blinded to the treatment paradigms. Survival periods are given in Supplementary Table S1.

Male C57BL6 mice (24–28 g) were exposed to 60 min of middle cerebral artery occlusion (MCAO) as previously described (Doeppner et al., 2017), followed by a reperfusion period for a maximum of 84 days. Briefly, mice received anesthesia with 1.5% isoflurane and underwent midline neck incision. The left common carotid artery (CCA) was prepared and after incision, a silicon-coated nylon monofilament (Doccol, United States) was gently inserted into the left CCA, which was then pushed forward through the internal carotid artery (ICA), reaching the proximal branch of the left middle cerebral artery (MCA). Once there, the filament was left *in situ* for 60 min under constant laser Doppler flow control. The body temperature was continuously measured using a rectal feedback probe and a heating pad, keeping the body temperature between 36.5°C and 37°C. Consequently, this setting allows for brain infarcts affecting the striatum and part of the cortex.

### Induction of rPostC and Experimental Groups

Induction of rPostC was essentially performed as previously described with some modifications (Ramagiri and Taliyan, 2017b). Non-invasive, rPostC was done using tourniquets for induction of transient ischemia of both hind legs. A complete cycle of rPostC consisted of three periods of a 10-min ischemia interrupted by 10 min of reperfusion of both hind legs. The experimental protocol of rPostC differed, depending on the survival periods of the animals. Mice that survived for 3 or 7 days, received their first rPostC at the time points given, i.e., at 12 h, at 24 h or at 120 h, followed by additional cycles of rPostC on each consecutive day until the time of sacrifice. For survival periods of 3 months, rPostC started at the time points given and was continued until day two (beginning of rPostC 12 h and 24 h only), whereas mice receiving their first cycle of rPostC on day five received additional cycles of rPostC on each consecutive day until day 14. For details please refer to Supplementary Figure S1.

### Analysis of Post-Ischemic Brain Injury

Brain injury at acute and subacute time points was assessed using triphenyltetrazolium chloride (TTC) staining on 2-mm-thick brain slices. In these slices, infarct volume was outlined and brain edema was calculated as relative increase of the ipsilateral compared to contralateral hemispheric volume. For long-term assessment of brain injury, animals were sacrificed on day 84 after MCAO for which mice were transcardially perfused with 4 % paraformaldehyde in 0.1 M phosphate-buffered saline (PBS). Thereafter, brains were removed, and 20 μm coronal cryostat sections were collected. The latter were used for immunohistochemistry for the neuronal marker NeuN, which was detected by a monoclonal mouse anti-NeuN antibody (1:1000; Millipore, United Kingdom). Quantitative analysis of the density of surviving neurons in the ischemic striatum was done within four regions of interest in three sections per animal at AP + 0.14 mm, ML ± 1.5–2.25 mm, and DV -2.5–3.25 mm from bregma.

### Analysis of Post-Stroke Neuroregeneration

Neuroregeneration as indicated herein by endogenous neurogenesis and angiogenesis, was analyzed 3 months after stroke induction. As such, mice received intraperitoneal injections of bromodeoxyuridine (50 mg/kg, Sigma-Aldrich, Germany) on days 8–28. Following transcardial perfusion with 4% paraformaldehyde in 0.1 M PBS on day 84, immunohistochemical analysis was performed in coronal cryostat sections using the following primary antibodies: monoclonal mouse anti-BrdU (1:400; Roche, Germany), monoclonal rat anti-BrdU (1:400; Abcam, United Kingdom), polyclonal goat anti-doublecortin (Dcx; 1:50; Santa Cruz Biotechnology) and monoclonal mouse anti-NeuN (1:1000; Millipore) and monoclonal rat anti-CD31 (1:200, BD Biosciences, Germany). Following detection with appropriate secondary antibodies, the density of proliferating BrdU^+^ cells, BrdU^+^/Dcx^+^ neurons, BrdU^+^/NeuN^+^ neurons, and BrdU^+^/CD31^+^ endothelial cells was evaluated as described for NeuN immunohistochemistry using the same coordinates and regions of interest.

### Assessment of Post-Stroke Neurological Recovery

All mice received training 1–2 days before MCAO in order to ensure proper test performance in the rota rod test, the tight rope test, the corner turn test, and the balance beam test. These tests were performed as previously described (Doeppner et al., 2014a). The rota rod test, the tight rope test, and the balance beam test were performed in duplicates per test day from which means were calculated. Readout in the rota rod test was the time until the animal dropped off the rotating drum with a maximal testing time of 300 s, whereas in the tight rope test the time until the animal reached the platform was assessed (maximal testing time 60 s). Test scores in the tight rope test ranged from 0 (minimum) to 20 (maximum). The balance beam test measured the time the animal needed to reach the platform by crossing a horizontal beam with continuously narrowing width (maximal testing time 60 s). In the corner turn test, the laterality index was calculated out of 10 trials per test day, with a score of 0.5 indicating no neurological impairment and a score of 1 indicating severe neurological impairment.

### Knockdown of Hsp70

For some experiments, stereotactic injections prior to induction of MCAO were performed in order to evaluate whether or not Hsp70 is involved in the aforementioned neuroprotection on day 3 induced by rPostC. As such, Hsp70 was knocked down using an Accell SMARTpool mouse Hsp70 small interfering RNA (siRNA) (4 mg/kg body weight; Dharmacon, Germany), which was intracerebrally administered into the striatum 3 days before stroke as previously described (Doeppner et al., 2012b). For control, mice received Accell non-targeting small interfering RNA (4 mg/kg body weight; Dharmacon) injections. Thereafter, rPostC was induced as explained afore, i.e., starting either 12 h or 24 h after stroke. Infarct volume analysis using TTC staining was performed on day 3 after MCAO.

### Measurement of Proteasome Activity

Assessment of proteasome activity was performed as described previously (Doeppner et al., 2012b). Briefly, proteasome activity was measured in brain homogenates of left (ischemic) hemispheres 72 h post-stroke using lysis buffer (100 mM *Tris-Hcl*, 145 mM NaCl, 10 mM EDTA, and 0.5% Triton X-100 at pH 7.5). The chymotrypsin-like activity of the proteasome was measured using Suc-LLVY-AMC (50 μM; Sigma-Aldrich, Germany) as a substrate that was incubated with 90 μl of reaction buffer containing 50 mM Tris, 20 mM KCl, 1 mM magnesium acetate, 2 mM dithiothreitol (DTT), 1 mM leupeptin, 1 μg/ml aprotinin (Sigma-Aldrich) and 1 mM PMSF (Merck, Germany). The extent of substrate cleavage was evaluated at 37°C in a fluorescence microtiter plate reader at λ_exc._ = 355 nm and at λ_em._ = 460 nm. Since Suc-LLVY is not exclusively cleaved by the proteasome, specificity was increased by adding the proteasome inhibitor MG-132 (1 mM; Sigma-Aldrich, Germany) at the beginning of the measurement. Proteasome activities were given as arbitrary units per min per mg protein. Protein concentration was measured using the Bradford assay.

### Growth Factor Measurements

The concentration of brain-derived neurotrophic factor (BDNF; Promega, Germany), glial cell line-derived neurotrophic factor (GDNF, Promega), vascular endothelial growth factor (VEGF; R&D Systems, United States), nerve growth factor (NGF; Promega), basic fibroblast growth factor (bFGF; R&D Systems) and epidermal growth factor (EGF; R&D Systems) was measured by enzyme linked immunosorbent assay (ELISA) in left (ischemic) hemispheres 84 days after MCAO as described before (Doeppner et al., 2012a).

### Analysis of Post-Stroke Inflammatory Responses

Flow cytometry analysis was performed on day 7 in order to assess post-ischemic immune responses both in the brain and in the blood as previously described (Chu et al., 2014; Doeppner et al., 2014b, 2015). Briefly, for analysis of blood samples, leukocytes were purified using lysis buffer consisting of 155 mM NH_4_Cl, 10 mM KHCO_3_ and 3 mM EDTA. For flow cytometry measurements of brain tissue samples, ischemic left hemispheres were mechanically homogenized in a buffer of collagenase type XI (125 U/ml), hyaluronidase (60 U/ml) and collagenase (450 U/ml) in Ca^2+^/Mg^2+^ supplemented PBS (Sigma-Aldrich, Germany). Samples were incubated with the antibody in question as described afore (Doeppner et al., 2015). Absolute cell numbers were measured using CountBright counting beads (Invitrogen, United States).

### Statistics

All data are given as mean ± standard deviation (SD). For comparisons between two groups, statistical comparisons were done using the Student *t* tests. For comparison between multiple groups, a one-way ANOVA followed by the Tukey’s *post hoc* test was used. *P*-values < 0.05 were considered to be statistically significant.

## Results

### Post-Stroke Neuroprotection by rPostC Depends on Delivery Timing

**FIGURE 1:**
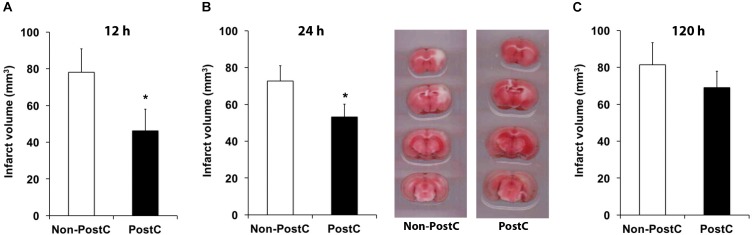
Remote post-conditioning (rPostC) induces acute neuroprotection when applied within 24 h post-stroke. rPostC was induced at 12 h **(A)**, at 24 h **(B)**, or at 120 h **(C)** after stroke, using the experimental protocol given in the materials and methods section. Analysis of infarct volumes is given for control animals (Non-PostC) and treated mice (PostC) using TTC staining on day 3 **(A,B)** and on day 7. Representative TTC stainings are given for groups using the 24 h experimental paradigm **(B)**. Data is given as mean ± standard deviation with ^∗^ indicating statistical significance (*p* < 0.05).

**FIGURE 2:**
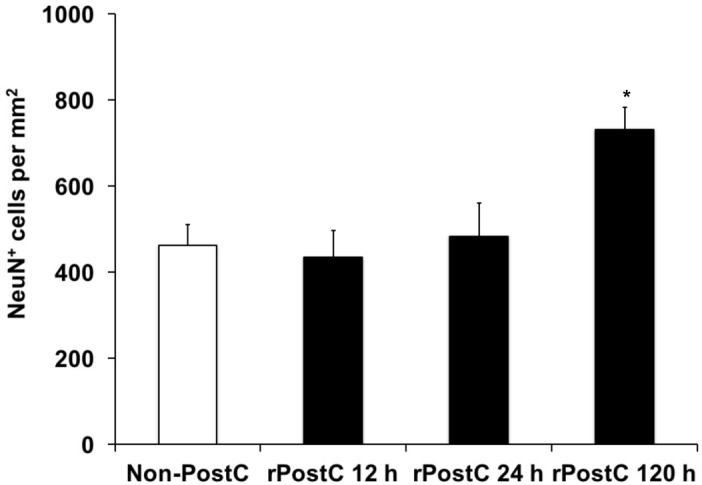
Sustained neuroprotection is a consequence of very delayed rPostC only. Long-term neuroprotection was analyzed using determination of neuronal density on day 84 after stroke induction as described in the materials and methods section. Time points given refer to the induction of rPostC, whereas control animals did not receive rPostC (Non-PostC). Data is given as mean ± standard deviation with ^∗^ indicating statistical significance compared to both Non-PostC and other rPostC groups (*p* < 0.05).

Experimental stroke paradigms analyzing the effect of rPostC on brain injury are usually restricted, as they apply one experimental paradigm with observation periods no longer than 2 weeks only. Hence, we systematically analyzed three different experimental paradigms that differed between the start of the first induction of rPostC. Indeed, early rPostC reduces infarct volumes 3 days after stroke when applied within 24 h after stroke onset (Figures 1A,B). The reduction of infarct volume after rPostC was profound and significant, i.e., infarct volumes were reduced by 39.8% (rPostC 12 h) and by 26% (rPostC 24 h), respectively. However, very delayed rPostC failed to reduce infarct volumes on day seven as applied 120 h after stroke onset (Figure 1C). Taking into account that the pathophysiology of stroke involves the development of secondary cell death, thus leading to the development of further brain injury even beyond acute and subacute stages of the disease, brain injury was analyzed 3 months after stroke induction as well. Interestingly, initial neuroprotection after early rPostC within 24 h after stroke onset (Figures 1A,B) was lost in the long run, i.e., neuronal density did not differ between controls and mice treated with rPostC in these groups (Figure 2). On the contrary, mice that were exposed to very delayed PostC 120 h after stroke induction showed sustained neuroprotection (Figure 2), albeit these mice did not show signs of subacute neuroprotection (Figure 1C). En detail, rPostC initiated at 120 h increased neuronal density by 60.1% 3 months after stroke induction.

### Sustained Neuroprotection due to Very Delayed rPostC Is Associated With Better Neurological Recovery

**FIGURE 3:**
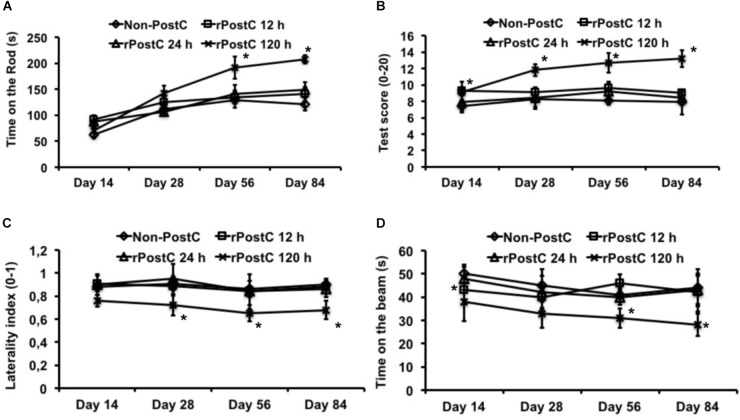
Sustained neurological recovery is achieved by very delayed rPostC only. Four different behavioral tests were used in order to assess post-stroke neurological outcome. These tests include the rota rod test **(A)**, the tight rope test **(B)**, the corner turn test **(C)**, and the balance beam test **(D)**. All animals were trained before induction of transient focal cerebral ischemia in order to ensure proper test performance at the time points given. rPostC was induced at 12 h, at 24 h, or at 120 h. Non-PostC mice did not receive post-stroke rPostC. Data is given as mean ± standard deviation with ^∗^ indicating statistical significance compared to both Non-PostC and other rPostC groups (*p* < 0.05).

Induction of early rPostC during the acute phase of the disease, i.e., 12 and 24 h after stroke onset, yields transient neuroprotection only that is lost during the observation period of 3 months (Figures 1, 2). Likewise, mice treated with early rPostC show a transient improvement of neurological recovery in the four behavioral tests only at the early stage of the disease (data not shown). In contrast to that, the aforementioned animals did not develop better test performance in the rota rod, the tight rope, the corner turn or the balance beam test (Figure 3). Mice that were treated with very delayed rPostC starting 120 h after stroke onset, however, gradually improved in the four behavioral tests during the observation period of 3 months (Figure 3). Consequently, very delayed rPostC induces sustained and increased neurological recovery, whereas early rPostC induces transient neurological recovery only.

### Neuroprotection Upon Induction of Early rPostC Involves Both Proteasomal and Heat Shock Protein 70 (Hsp70) Cell Signaling

**FIGURE 4:**
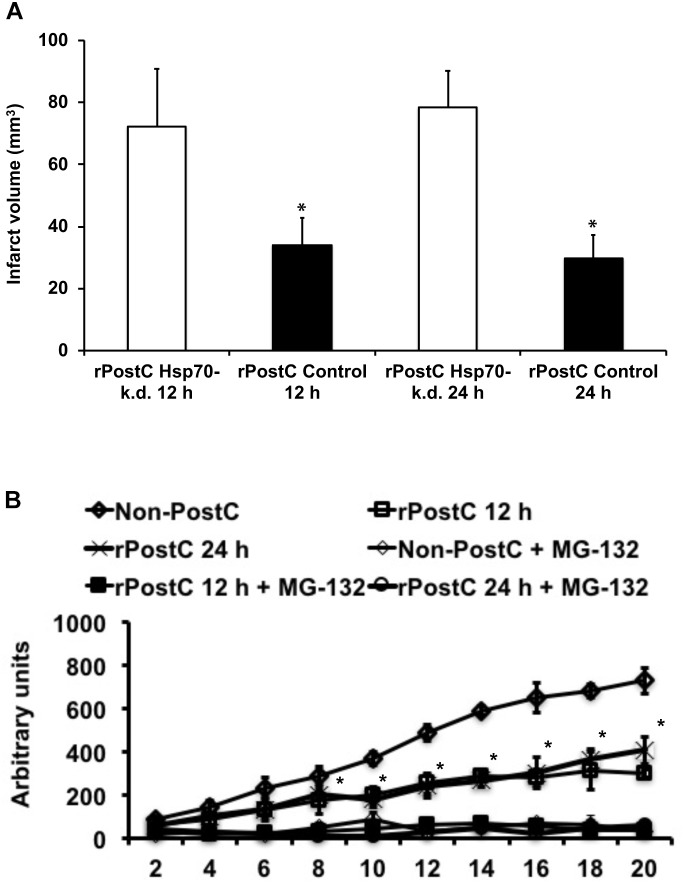
Acute neuroprotection upon rPostC involves the Hsp70-proteasome pathway. **(A)** Mice received stereotactic injections in the future ischemic striatum 3 days before induction of stroke. Injections either contained an Accell SMARTpool mouse Hsp70 small interfering RNA (rPostC Hsp70-k.d.) or an Accell non-targeting small interfering RNA (rPostC Control). Thereafter, animals underwent induction of stroke followed by exposure to rPostC at 12 h or at 24 h post-stroke. Infarct volume analysis was done 3 days after stroke induction using TTC staining. **(B)** Determination of proteasomal chymotrypsin-like activity from brain lysates of the ischemic hemisphere 3 days after stroke using Suc-LLVY-AMC as substrate. Suc-LLVY-AMC is not a specific proteasome substrate. Therefore, some samples were incubated with the proteasome inhibitor MG-132 (1 mmol/L) immediately before the measurement in order to ensure recording of proteasomal activity. Data are given as arbitrary fluorescence units per minute per mg of total protein. ^∗^Significantly different (*p* < 0.05).

The pathophysiology of stroke involves inflammatory signaling cascades (Dirnagl et al., 1999), such as heat shock proteins that act as physiological ligands of toll-like-receptors (TLR) that regulate pro-inflammatory signaling cascades including the ubiquitin-proteasome-pathway (Vabulas et al., 2002; Asea, 2008; Tamura et al., 2012; Napetschnig and Wu, 2013). Whereas previous data has described a role for TLR being involved in ischemic post-conditioning, a role for Hsp70 in direct cerebral post-conditioning has recently been shown by our group for the very first time (Feng et al., 2011; Wang et al., 2014; Doeppner et al., 2017). Herein, we demonstrate that Hsp70 is critically involved in mechanisms underlying rPostC after focal cerebral ischemia as well. Knocking down of Hsp70 by means of siRNA constructs that had been injected 3 days before induction of stroke with subsequent rPostC, infarct volumes were significantly increased on day 3 post-stroke when compared to mice that had received control non-targeting siRNA constructs (Figure 4A). As such, infarct volumes were increased by 100.8% (induction of rPostC at 12 h) and by 147.0%, respectively.

Along with the aforementioned role of Hsp70 in the herein used experimental paradigm, proteasomal activity was significantly altered by induction of early rPostC (Figure 4B). As such, induction of early rPostC significantly reduced the chymotrypsine-like activity of the proteasome in comparison to mice that were not treated with rPostC. This data suggests that inhibition of the proteasome might be one factor, leading to rPostC-induced neuroprotection.

### Very Delayed Induction of rPostC Induces Increased Post-Stroke Neuroregeneration and Modification of the Post-Stroke Extracellular Milieu

**FIGURE 5:**
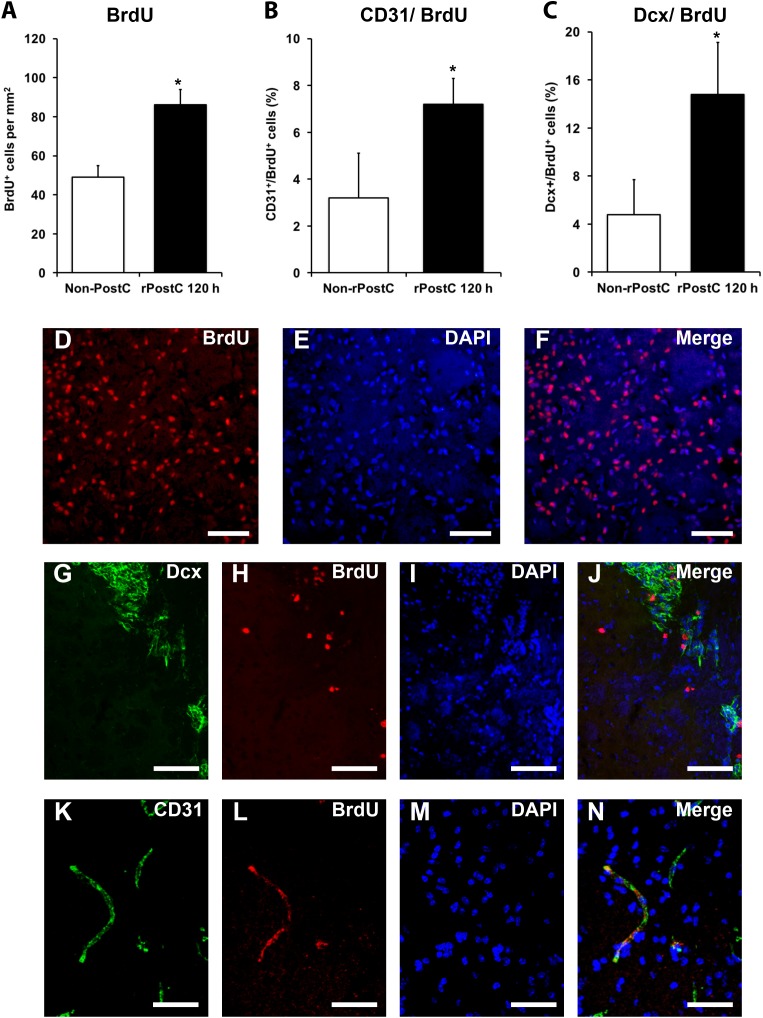
Very delayed rPostC is associated with increased neuroregeneration. Quantitative analyses of bromodeoxyuridine positive (BrdU^+^) proliferating cells **(A)** as well as the relative co-expression of the endothelial marker CD31 **(B)** and the immature neuronal marker Dcx **(C)** on day 84 in ischemic mice that were exposed to rPostC 120 h after stroke induction. Mice that were not exposed to rPostC (Non-PostC) served as control. Representative photos taken from within the ischemic lesion site for BrdU staining **(D–F)**, Dcx staining **(G–J)** and CD31 staining **(K–N)**. Scale bars: 100 μm. ^∗^Significantly different from Non-PostC, (*p* < 0.05).

In contrast to early rPostC, very delayed rPostC induces sustained neurological recovery and long-term neuroprotection without being neuroprotective at subacute stages of the disease (Figures 1, 2), suggesting different mechanisms being involved depending on the timing of induction of rPostC. As such, we next addressed the question whether or not very delayed rPostC affects endogenous neurogenesis, which is known to persist in the adult mammalian brain as well (Alvarez-Buylla and Garcia-Verdugo, 2002). Indeed, induction of very delayed rPostC resulted in significantly increased numbers of BrdU^+^ proliferating cells within the ischemic hemisphere (Figures 5A,D-F). Likewise, an analysis of co-expression patterns of these BrdU^+^ cells revealed that rPostC yielded increased co-expression of BrdU^+^ cells with both the endothelial marker CD31 and the immature neuronal marker Dcx 3 months after stroke (Figures 5B,C,G-N).

**FIGURE 6:**
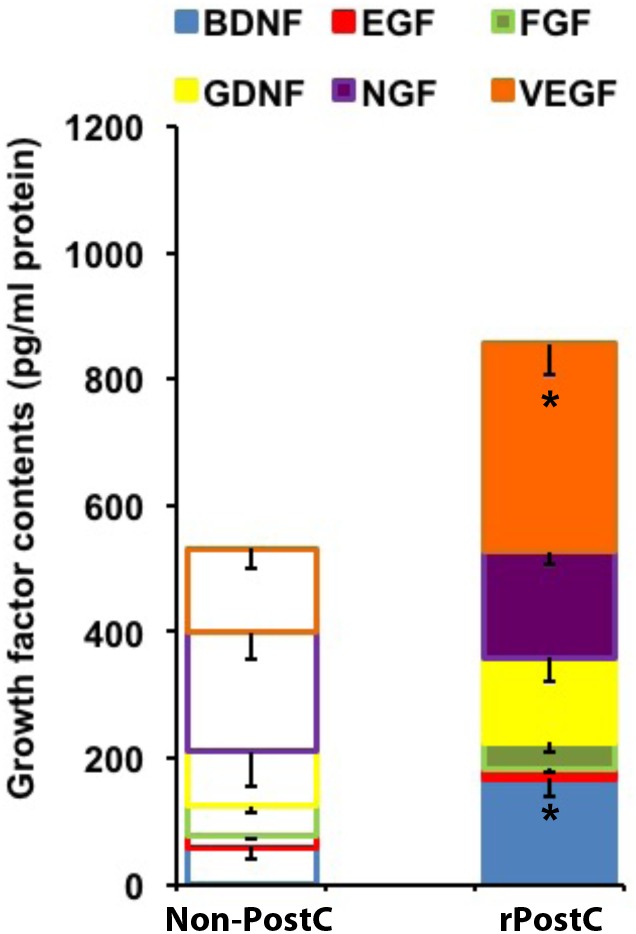
Very delayed rPostC modifies the post-stroke extracellular milieu. The concentration of selected growth factors was measured in ischemic hemispheres on day 84 post-stroke. Mice were exposed to rPostC at 120 h after stroke induction with mice receiving no rPostC (Non-PostC) serving as controls. ^∗^Significantly different from Non-PostC (*p* < 0.05).

Angiogenesis and neurogenesis are not independent from each other, but rather mutually affect each other (Hermann and Chopp, 2012), and stroke itself stimulates endogenous neurogenesis (Arvidsson et al., 2002), albeit the majority of new-born cells is not integrated in residing neural networks but undergoes secondary cell death (Doeppner et al., 2012a). In this context, increased numbers of immature neuronal cells co-expressing BrdU and Dcx after rPostC (Figures 5C,G-J) with no relevant co-expression of BrdU and the mature neuronal marker NeuN (0.45 ± 0.08% for Non-rPostC vs. 0.38 ± 0.19% for rPostC started at 120 h) is in line with previous observations. Rather, the aforementioned neuroprotection and neurological recovery observed after late rPostC is a consequence of indirect effects of new-born cells. NPCs and derivatives of them act like “mini pumps,” constantly secreting growth factors and other mediators, thus affecting their extracellular milieu (Doeppner et al., 2012a). Indeed, induction of very delayed rPostC resulted in significantly increased concentration of growth factors such as VEGF and BDNF, which were increased by 172 % (BDNF) and by 147.8% (VEGF), 3 months after stroke induction (Figure 6).

### Very Delayed rPostC Reverses Post-Stroke Immunosuppression

**FIGURE 7:**
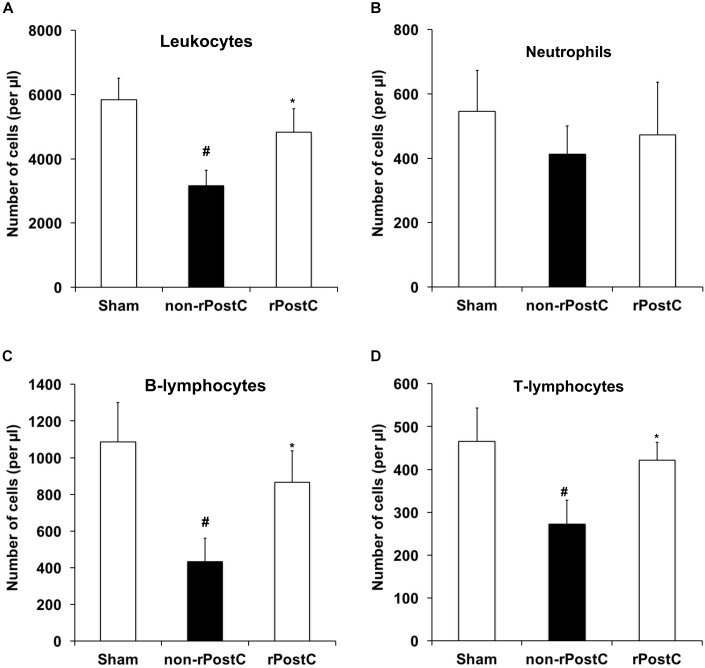
Very delayed rPostC reverses post-stroke peripheral immunosuppression. Flow cytometry analysis from blood samples was performed 7 days after stroke induction in mice that had received rPostC 120 h after stroke or in control mice that had not received rPostC (non-rPostC). Sham animals underwent surgical procedure for transient focal cerebral ischemia without inserting of the filament into the left middle cerebral artery. A quantitative analysis of leukocytes **(A)**, neutrophils **(B)**, B-lymphocytes **(C)**, and T-lymphocytes **(D)** was performed as described in the materials and methods section. ^∗^Significantly different from non-rPostC, (*p* < 0.05). ^#^Significantly different from Sham, (*p* < 0.05).

Very delayed rPostC affects post-stroke neuroregeneration and modulates the extracellular milieu (Figures 5, 6). However, stroke implies a plethora of signaling cascades among which both pro-inflammatory and anti-inflammatory signaling cascades are activated in a temporal resolution. Whereas stroke is well known to activate pro-inflammatory signaling cascades (Dirnagl et al., 1999), stroke itself is also known to induce immunosuppression at later stages of the disease (Dirnagl et al., 2007; Meisel and Meisel, 2011), affecting clinical outcome of stroke patients. In light of this, we wondered whether or not very delayed rPostC might affect stroke-induced immunosuppression. Seven days after stroke, induction of transient focal cerebral ischemia resulted in reduction of leukocyte numbers in the blood when compared to non-ischemic sham mice (Figure 7A). Induction of rPostC, however, reversed the stroke-induced immunosuppression on day 7. Further analysis revealed that rPostC predominantly affected the restoration of both T-lymphocytes and B-lymphocytes (Figures 7C,D). As such, rPostC increased the number of B-lymphocytes and T-lymphocytes by 112.7% and by 50.7%, respectively, when compared with ischemic controls, whereas neutrophils were not affected by rPostC.

## Discussion

The present study underlines the importance of application timing for hind limb rPostC in a mouse model of transient focal cerebral ischemia. Acute neuroprotection as observed after application of rPostC within 24 h (early rPostC) is mediated via Hsp70 which in turn inhibits proteasomal activation, yielding transient reduction of brain injury. On the contrary, very delayed rPostC reverses peripheral post-stroke immunosuppression which implies a modulation of the extracellular milieu, stimulating neuroregenerative processes that orchestrate sustained neurological recovery.

Direct ischemic post-conditioning has been shown to induce both neuroprotection and neurological recovery in a variety of experimental stroke models, including focal and global cerebral ischemia (Burda et al., 2006; Gao et al., 2008; Pignataro et al., 2008; Ren et al., 2008; Xing et al., 2008; Zhou et al., 2011; Xie et al., 2013). Although the majority of work published focuses on studying effects of ischemic cerebral post-conditioning when applied within the first hours after stroke onset, some data support the hypothesis that ischemic post-conditioning might still be effective when applied as late as 2 days after stroke onset. Indeed, previous work of our own demonstrates that ischemic cerebral post-conditioning is neuroprotective when applied within a critical period of time of 24 h post-stroke (Doeppner et al., 2017). However, ischemic direct post-conditioning fails to induce sustained neuroprotection in this model of transient focal cerebral ischemia. Rather, post-conditioning induces subtle modifications of the extracellular milieu which offers the possibility of successful transplantation of NPCs, thus ensuring sustained neurological recovery. Despite these promising results in the field of ischemic post-conditioning of the brain, the concept of direct ischemic post-conditioning provides little therapeutic benefit for an organ such as the brain. As a matter of fact, direct ischemic post-conditioning of the human brain, i.e., the induction of a second subinjurious ischemic stimulus via transient obliteration of the carotid arteries, is out of the question. Ischemic rPostC therefore appears to be more attractive than common direct ischemic post-conditioning of the brain from a clinical point of view.

Not only in light of its potential therapeutic relevance but also due to its practicability has ischemic rPostC been thoroughly studied in a variety of experimental models. The latter implies experimental studies on the kidney, the liver, the lung, the heart, and the brain, to name but a few (Ren et al., 2011; Hasseldam et al., 2013; Khan et al., 2015; Leung et al., 2015; Li P. et al., 2015; Gao et al., 2016, 2017; Ramagiri and Taliyan, 2017a,b; Sandweiss et al., 2017; Kitagawa et al., 2018; Ren et al., 2018). The mechanisms being involved are as diverse as the models that have been applied. Yet, it appears to be evident that well-known survival pathways as well as anti-oxidative and anti-inflammatory cascades are modulated by rPostC in this heterogeneous study group. In this sense, rPostC activates survival signaling pathways such as Akt, JAK/STAT3, HO-1, and GSK3beta, whereas the inhibition of at least some of these aforementioned signaling pathways reverses the rPostC-induced cellular protection (Li P. et al., 2015; Gao et al., 2016, 2017; Ramagiri and Taliyan, 2017a,b). As mentioned afore, however, these studies cannot easily be compared between each other as they significantly differ in their experimental models chosen, which does not only affect the organ in question but also the different timing chosen for the induction of rPostC.

The precise cellular and molecular mechanisms that are involved are neither known for pre-conditioning, per-conditioning, or post-conditioning of the brain, may it be direct conditioning or rPostC. Nevertheless, a great deal of evidence suggests that these different concepts of conditioning share some general signaling pathways (Zhao et al., 2012). Indeed, early rPostC within 24 h after stroke induction yields reduction of infarct volume, as has also been shown for direct post-conditioning by our group before (Doeppner et al., 2017). This data is in line with previous reports, showing that induction of rPostC within the very first hours after stroke onset is associated with reduction of acute brain injury (Ren et al., 2011; Hasseldam et al., 2013; Li P. et al., 2015; Ramagiri and Taliyan, 2017b). Of note, the reduction of infarct volume due to rPostC within 24 h as shown in the present study, exceeds rPostC intervals as applied in the aforementioned studies significantly. In this context, the acute neuroprotection in our experimental setting is mediated via Hsp70 and the ubiquitin-proteasome-system (UPS). Heat shock proteins such as Hsp70 are physiological ligands of toll-like receptors (TLRs) such as TLR 2 and TLR4 (Vabulas et al., 2002; Asea, 2008; Tamura et al., 2012), which in turn regulate pro-inflammatory signaling cascades among which are NF-kB and the UPS (Gilmore and Wolenski, 2012; Napetschnig and Wu, 2013). The role of Hsp70 in particular under experimental settings of direct cerebral post-conditioning, however, has been described by us recently (Doeppner et al., 2017). It is therefore not surprising that rPostC involves a similar mechanism which is herein shown for the first time, as indicated by both reduced proteasomal activity and reversal of rPostC-induced neuroprotection due to Hsp70 knockdown.

Induction of early rPostC does not yield sustained neuroprotection during the observation period of 3 months which is in line with previous data from our group, demonstrating that direct cerebral post-conditioning induces transient neuroprotection only (Doeppner et al., 2017). Likewise, previous studies on rPostC after experimental stroke have focused on short time survival periods only as described afore (Li P. et al., 2015; Gao et al., 2016, 2017; Ramagiri and Taliyan, 2017a,b). Whether or not neuroprotection observed in these studies is transient only remains elusive. On the contrary, very delayed rPostC induces sustained neuroprotection and improved neurological recovery as indicated by a battery of various well established behavioral tests (Doeppner et al., 2014a), which has not been described elsewhere under these conditions. Improved neurological recovery, however, is not a consequence of acute neuroprotection which was not observed under these conditions at all. Rather, very delayed rPostC induces pleiotropic effects which orchestrate the aforementioned neurological recovery and which has not been described in the literature elsewhere. This is in contrast to previous data from our own, demonstrating transient neuroprotection after direct ischemic post-conditioning only (Doeppner et al., 2017). Nevertheless, survival periods in that study were limited to 4 weeks only, showing no stimulation of endogenous neuroregeneration which might explain the discrepancy. As a matter of fact, very delayed rPostC modulates peripheral post-stroke immune responses which has been described before in a model of ischemic remote pre-conditioning (Liu et al., 2016), leading in our present study to a reversal of post-stroke immunosuppression. The latter is known to affect the post-ischemic extracellular milieu (Doeppner et al., 2015), which is observed in our study. The modification of the extracellular milieu, in turn, favors a survival of endogenous endothelial and neuronal progenitor cells (Doeppner et al., 2012a). Indeed, an enhanced post-stroke endogenous angioneurogenesis after very delayed rPostC helps orchestrate tissue regeneration via indirect bystander effects of newly generated cells, albeit these new-born cells are not integrated into residing neural networks. Rather, the constant secretive activity of these new-born cells helps boost post-stroke tissue regeneration. In line with this, recent work from Ren et al. (2018) has shown that angiogenesis is stimulated upon a combinative approach of both remote per-conditioning and rPostC after stroke, which is contributed to the Notch signaling pathway. Nevertheless, survival periods were limited to 2 weeks only, giving no information regarding the contribution of angiogenesis to neurological recovery in the long run.

The concept of remote conditioning in general and the concept of rPostC in particular are interesting concepts for adjuvant treatment paradigms of different diseases. Indeed, first clinical trials have been performed, suggesting possible beneficial effects after remote conditioning (Zuo et al., 2015; Bei et al., 2016; Hou et al., 2016; England et al., 2017; Wang et al., 2017; Zhao et al., 2017; Zhou et al., 2017). Yet, the study concepts are heterogeneous, including small study populations with special medical conditions only. Leaving aside clinical trials on various settings of renal malfunction (Bei et al., 2016; Zuo et al., 2015; Zhou et al., 2017) as well as trials on chronic vessel diseases of the brain (Hou et al., 2016; Wang et al., 2017; Zhao et al., 2017), the RECAST study remains the only clinical trial where rPostC has been applied as a therapeutic means to counter a territorial ischemic stroke during the acute phase of the disease (England et al., 2017). Of note, rPostC in the RECAST trial like in the RESCUE BRAIN trial, which is currently recruiting (Pico et al., 2016), treatment starts within 24 h post-stroke. According to the findings of the present study, however, acute rPostC results in transient neuroprotection, whereas very delayed rPostC only yields a significant impact on the post-stroke neurological outcome in the long-run. The choice for the optimal therapeutic time frame of post-stroke rPostC in future clinical trials might therefore be reconsidered. Consequently, further pre-clinical studies on rPostC after stroke are urgently needed in order to further validate the importance of very delayed rPostC, thus avoiding negative and ill-designed future clinical trials.

## Author Contributions

TD designed and reasoned the study, performed the experiments, wrote the manuscript, and analyzed the data. BZ performed the experiments and analyzed the data. BK, FJ, XZ, and VV performed the experiments. AM designed and reasoned the study. MB and DH designed and reasoned the study, provided financial support, and wrote the manuscript.

## Conflict of Interest Statement

The authors declare that the research was conducted in the absence of any commercial or financial relationships that could be construed as a potential conflict of interest.
