# Supplementary material for: Very Delayed Remote Ischemic Post-conditioning Induces Sustained Neurological Recovery by Mechanisms Involving Enhanced Angioneurogenesis and Peripheral Immunosuppression Reversal
Source: Front Cell Neurosci. 2018 Oct 29;12:383. doi: 10.3389/fncel.2018.00383 (PMC6216109; doi:10.3389/fncel.2018.00383)
Supplement: Supplementary file 1 [file Data_Sheet_1.PDF]

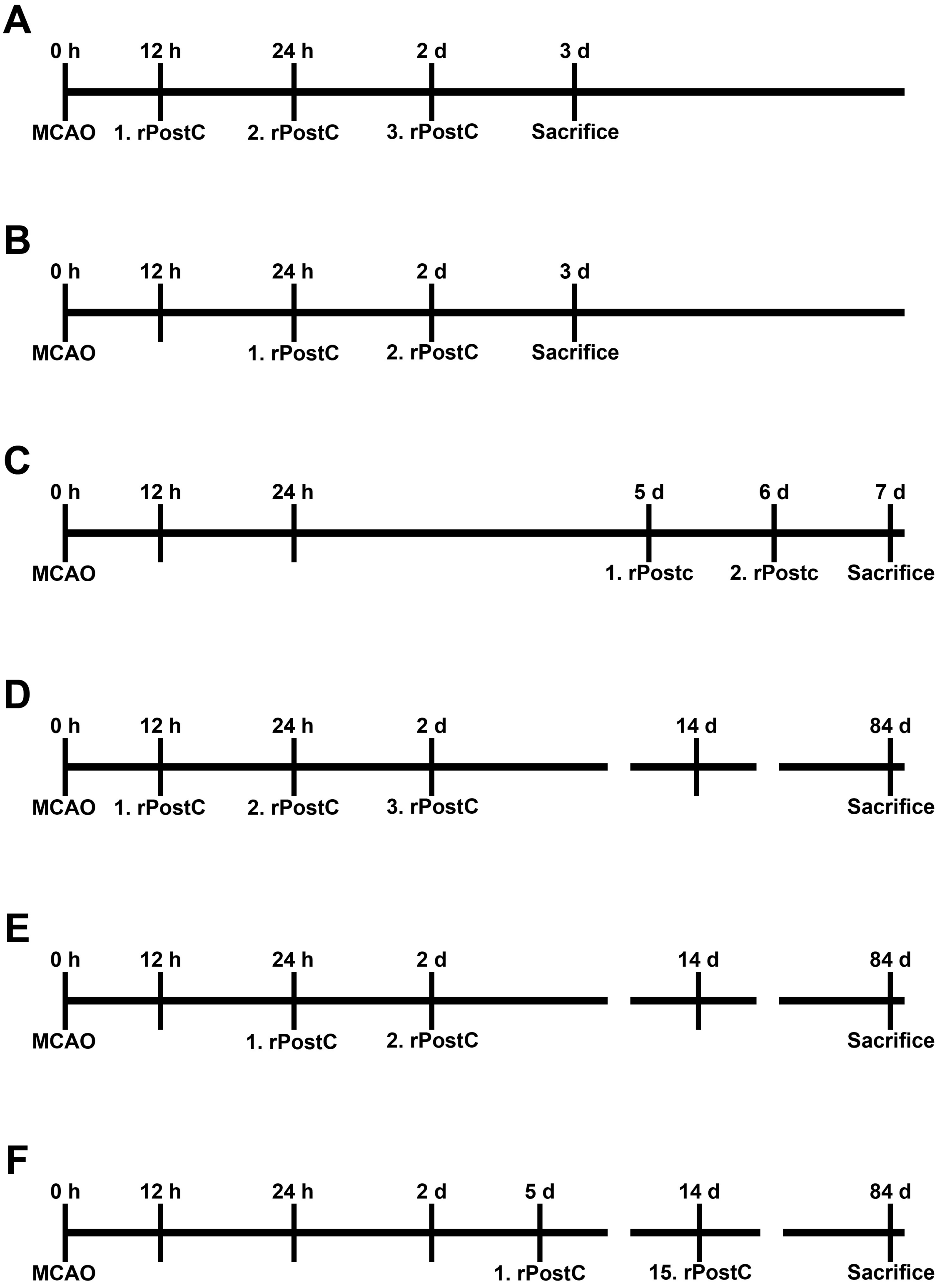
A
0 h
12 h
24 h
2 d
3 d
MCAO
1. rPostC
2. rPostC
3. rPostC
Sacrifice
B
0 h
12 h
24 h
2 d
3 d
MCAO
1. rPostC
2. rPostC
Sacrifice
C
0 h
12 h
24 h
5 d
6 d
7 d
MCAO
1. rPostc
2. rPostc
Sacrifice
D
0 h
12 h
24 h
2 d
14 d
84 d
MCAO
1. rPostC
2. rPostC
3. rPostC
Sacrifice
E
0 h
12 h
24 h
2 d
14 d
84 d
MCAO
1. rPostC
2. rPostC
Sacrifice
F
0 h
12 h
24 h
2 d
5 d
14 d
84 d
MCAO
1. rPostC
15. rPostC
Sacrifice

Supplementary Figure S1. Experimental treatment paradigm for remote ischemic post-conditioning (rPostC). All mice were exposed to middle cerebral artery occlusion (MCAO). Mice that were allowed to survive for 3 days received their first cycle of rPostC either 12 h **(A)** or 24 h **(B)** after induction of MCAO followed by additional rPostC on until day 2. For mice that were allowed to live for as long as 7 days **(C)**, rPostC was induced 5 days after MCAO with an additional cycle of rPostC on day 6. When mice were sacrificed on day 84, some animals underwent the aforementioned cycles of rPostC starting 12 h **(D)** or 24 h **(E)** after induction of MCAO. On the contrary, the experimental protocol for mice receiving their first cycle of rPostC on day 5 was significantly changed **(F)**. These mice received additional cycles of rPostC on each consecutive day until day 14.
