# Supplementary material for: Very Delayed Remote Ischemic Post-conditioning Induces Sustained Neurological Recovery by Mechanisms Involving Enhanced Angioneurogenesis and Peripheral Immunosuppression Reversal
Source: Front Cell Neurosci. 2018 Oct 29;12:383. doi: 10.3389/fncel.2018.00383 (PMC6216109; doi:10.3389/fncel.2018.00383)
Supplement: Supplementary file 2 [file Data_Sheet_2.PDF]

Supplementary Table S1. Experimental animals used including survival periods.

	day 3	day 7	day 84
**rPostC 12 h**	n=7 (100 %)	n. a.	n=16 (72.7 %)
**Control 12 h**	n=8 (100 %)	n. a.	n.a.
**rPostC 24 h**	n=5 (83.3 %)	n. a.	n=20 (95.2 %)
**Control 24 h**	n=7 (100 %)	n. a.	n.a.
**rPostC 120 h**	n. a.	n=15 (100 %)	n=24 (83.9 %)
**Control 120 h**	n.a.	n=16 (100 %)	n= 26 (81.3 %)
**Sham**	n.a.	n=7 (100 %)	n.a.

For all experiments, male C57BL6 mice were used that were sacrificed either on day 3, 7 or 84. For some groups, significantly more animals were needed as different readout parameters were measured that required processing of samples in different ways. Sham mice underwent surgery for induction of stroke, but neither received cerebral ischemia nor rPostC. Figures given in brackets refer to survival rates. The number of animals mentioned refers to the number of animals that have been actually used per experimental group per time point given. Abbreviation: rPostC: remote post-conditioning.
